# The Impact of COVID-19 on the Neck of Femur Fracture Service in a Tertiary Care Hospital in the United Kingdom

**DOI:** 10.7759/cureus.47298

**Published:** 2023-10-18

**Authors:** Upamanyu Nath, Benyamin Alam, Abhirun Das, Abdelwakeel Bakhiet, Anand Pillai

**Affiliations:** 1 Trauma and Orthopedics, Wythenshawe Hospital, Manchester University NHS Foundation Trust, Manchester, GBR

**Keywords:** covid-19, neck of femur (nof) fracture, frail elderly, elderly patients, covid-19 lockdown, ortho surgery

## Abstract

Introduction

The emergence of the novel coronavirus, severe acute respiratory syndrome coronavirus 2 (SARS-CoV-2), in late 2019 ushered in a global crisis that profoundly impacted healthcare systems worldwide. In the United Kingdom, COVID-19 resulted in a significant toll on public health and the National Health Service (NHS). As the virus surged, the NHS faced unprecedented challenges, including surges in COVID-19 cases, a dire need for medical equipment, and a strain on intensive care units. Simultaneously, stringent nationwide lockdowns were imposed to curb the virus's spread, disrupting daily life and healthcare access. Amid this crisis, the interactions between COVID-19 and other prevalent health conditions came to the forefront of medical research, sparking interest in understanding their connections. This study delves into the intriguing interplay between COVID-19 and neck of femur (NoF) fractures, exploring shared risk factors, resource implications, and potential alterations in patient pathways. Given the severity of both conditions and their impact on the vulnerable elderly population, elucidating these connections is crucial for comprehensive patient care and resource allocation within the healthcare system.

Methods

This study used data from the National Hip Fracture Audit (NHFA) database, focusing on NoF fracture patients at Wythenshawe Hospital. We examined two cohorts: pre-pandemic (from March 2019 to March 2020) and pandemic (from March 2020 to March 2021). We compared key parameters and incorporated COVID-19 data. Graphs showed trends and cohort similarities. We also analyzed demographic data (age, gender, fracture type, times, COVID-19 status, and mortality), removing outliers for accuracy.

Results

The data revealed that while certain factors such as patient age and mobilization remained largely unaffected, there was a modest association between COVID-19 incidence and NoF fracture patients. Notably, regional lockdown measures had a substantial impact on patient care. The initial lockdown effectively reduced COVID-19-positive cases upon admission but led to prolonged intervals and surgical delays. However, the second lockdown showed improvements, attributed to lessons learned, increased resource allocation, and better familiarity with hospital-specific lockdown measures. This research sheds light on the intricate relationship between a global pandemic and orthopedic patient care, highlighting the importance of adapting healthcare systems to evolving challenges.

Conclusion

This study explores the impact of COVID-19 on neck of femur (NoF) fracture patients, highlighting key findings from Wythenshawe Hospital. It uncovers a dynamic relationship between the pandemic and patient care, with increased COVID-19 cases coinciding with reduced NoF fracture rates. Lockdowns influenced outcomes, with the first causing delays and higher post-discharge mortality, while the second improved efficiency and safety. These insights extend beyond Wythenshawe Hospital, offering implications for healthcare practices in the United Kingdom and beyond, especially in countries with limited vaccination resources. This research underscores the need for tailored strategies to optimize NoF fracture patient outcomes during pandemics and lockdowns.

## Introduction

In December 2019, the novel coronavirus, known as severe acute respiratory syndrome coronavirus 2 (SARS-CoV-2) or COVID-19, was first identified in the United Kingdom [1]. This highly transmissible virus spread rapidly throughout the country, resulting in over four million positive tests and 124,000 deaths within 15 months [2]. While COVID-19 cases were observed across all demographics, individuals who were elderly, frail, immunocompromised, or had multiple comorbidities faced an increased risk of contracting COVID-19 and experiencing severe outcomes [3]. This had profound repercussions on both the UK population and the National Health Service (NHS).

The NHS faced unprecedented demands for medical equipment, including ventilators, oxygen therapy, and personal protective gear for both patients and healthcare workers. The surge in COVID-19 cases also led to a substantial need for intensive care unit space [4]. Consequently, the United Kingdom implemented nationwide lockdowns, restricting non-essential activities and work. These restrictions, while essential for controlling the virus's spread, had significant effects on public physical and mental health due to reduced physical activity and drastic lifestyle changes [5]. Furthermore, general practices (GPs) became less accessible than previously, with the NHS.uk website stating, "You'll only be asked to visit the surgery if absolutely necessary" [6]. Therefore, the COVID-19 pandemic had far-reaching implications for various health conditions, affecting incidence rates, mortality, and the quality of hospital care.

Neck of femur (NoF) fractures are a significant medical issue throughout the United Kingdom, predominantly affecting older individuals. NoF fractures are the most common serious injuries among older adults, often requiring emergency surgery and anesthesia [7]. Patients with these fractures typically have longer in-patient stays, with approximately one in 45 hospital beds occupied by hip fracture patients in 2019 [7]. These fractures are more prevalent in the elderly and are associated with various risk factors, such as low mobility, female sex, vitamin D deficiency, falls, comorbidities, corticosteroid use, and trauma [8]. Given the systemic nature of these risk factors and the comprehensive impact on patients' quality of life, a multidisciplinary approach involving various healthcare professionals is crucial for holistic patient care.

Notably, there is considerable overlap between COVID-19 and NoF fractures in terms of shared risk factors [9]. The demographic most susceptible to NoF fractures is also at a higher risk of contracting COVID-19 [10]. Moreover, both conditions impose substantial demands on NHS resources, potentially affecting the quality of care for NoF fracture patients during their hospitalization. Lockdown measures may have influenced the incidence of NoF fractures by promoting a sedentary lifestyle, which could lower mobility and lead to vitamin D deficiency, both risk factors for these fractures. However, lockdowns may have also reduced the probability of falls by limiting non-essential activities [11]. Consequently, a theoretical correlation exists between COVID-19 and NoF fractures, as the virus may have altered the patient pathway for these fractures.

## Materials and methods

Aims

The aim of this study is to assess the extent of the correlation between COVID-19 and NoF and identify whether COVID-19 has had any impact on NoF fractures in clinical practice within Wythenshawe Hospital. Elucidating the association between the two can enhance the understanding of the virus and its impact on the United Kingdom.

Literature review

The limited literature on the impact of COVID-19 on patients with NoF fractures presents conflicting evidence, emphasizing the need for more comprehensive research. Wang et al.'s systematic review investigated early mortality risk in COVID-19-positive NoF fracture patients undergoing surgery, revealing a fivefold increase in mortality risk, especially among those with comorbidities such as obesity [12]. However, this study solely focused on direct mortality without considering broader effects such as incidence, demographic changes, or quality of care. To gain a more holistic understanding, future research should adopt a broader perspective.

Rasidovic et al.'s multicenter cohort study in the United Kingdom during the pandemic's early phases showed a marked increase in mortality among COVID-19-positive NoF fracture patients (32.5% versus 7.2% in COVID-19-negative patients) [13]. Nevertheless, this study also concentrated solely on mortality and did not explore other variables or the evolving pandemic context. The introduction of personal protective equipment (PPE), increased testing, and vaccination distribution could potentially alter these findings.

Conversely, Craig et al.'s audit of hip fracture management and mortality during the pandemic in a regional trauma center found the maintenance of service efficacy and similar outcomes compared to pre-pandemic data [14]. The study indicated improvements in surgical timing despite reduced theatre capacity. However, this audit spanned only a two-month period during the lockdown, and outcomes may differ over a longer period and with changing restrictions and public attitudes. Additionally, being conducted in an Irish regional facility, differences from the United Kingdom's experience are likely.

Williams and Kumar's cohort study showed a 64% increase in admission-to-surgery time during and after the introduction of COVID-19 contingency plans [15]. Like Craig et al.'s [14] study, it has limitations due to its small sample size and a short observation period within the evolving pandemic context.

In summary, existing research on the impact of COVID-19 on NoF fracture patients is scarce, specific, and often contradictory. Clear conclusions are challenging to draw. To better understand the consequences of COVID-19 on these patients' health, further research is imperative for future public health considerations.

Methodology

Data was collected from the National Hip Fracture Audit (NHFA) database for NoF fracture patients from two separate cohorts within Wythenshawe Hospital. One dataset contained all patients who presented with a neck of femur fracture in the year prior to the pandemic between 18 March 2019 and 17 March 2020. The other dataset comprised all patients who presented with neck of femur fractures during the COVID-19 pandemic, from 18 March 2020 to 17 March 2021. Certain parameters were collected from each dataset for comparison. The final dataset consisted of the government website for the incidence of COVID-19 between 18 March 2020 and 17 March 2021; this acted as a marker of the impact of the virus on population health, burden on healthcare services, and public opinion toward COVID-19 during the year. For each parameter, a graph was created, within which both trends from the NHFA cohorts were plotted alongside the incidence of COVID-19. This allowed for insight into whether the March 2020/2021 NoF cohort mirrored the COVID-19 pattern or the previous NoF more.

The following parameters were collected from both NHFA cohorts: incidence, delay in theatre, delay in mobilization, and mortality. The March 2020/2021 cohort was compared against both the March 2019/2020 cohort and the national incidence of COVID-19 to demonstrate any trends and, if present, which dataset they followed more closely. Further analysis of changes during periods of lockdown was conducted to identify any impact this might have had.

Additional demographic data was collected from the March 2020/2021 cohort including patient age at presentation, patient sex, type of fracture, time from presentation to admission, time from presentation to theatre, time from presentation to discharge, patients testing positive for COVID-19, and positive patient mortality. These were plotted and compared against COVID-19 incidence to investigate any variations throughout the year and during lockdown, with outliers removed to prevent skew.

## Results

Comparing 2020/2021 data to see a correlation with 2019/2020 data and COVID-19 data

Incidence of NoF Fractures

**Figure 1:**
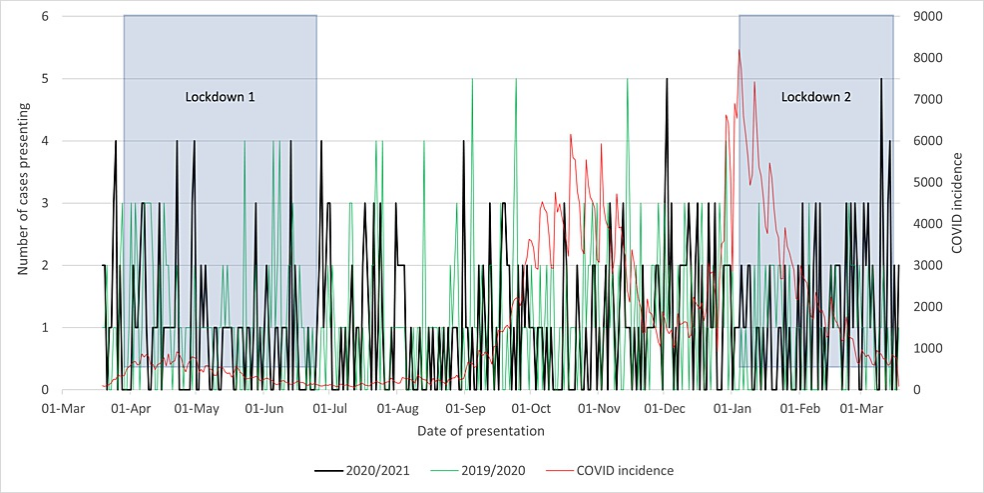
Cases of neck of femur fractures presenting in 2020/2021 compared to that in 2019/2020 and to the incidence of COVID-19 cases and lockdowns

The analysis of graph data from Figure 1 shows that the March 2020/2021 cohort and the March 2019/2020 cohort at Wythenshawe Hospital were observed to have a similar pattern, deviating from the COVID-19 incidence trend. Throughout the year, there were no significant differences in patient presentations to the emergency department. However, a minor 4.23% decrease in presentations was noted. Although the statistical significance of this slight variation may be debatable, it could be linked to the regional lockdown measures imposed in the area. These restrictions may have led to individuals spending more time at home, potentially reducing their risk of falls, a common factor contributing to neck of femur fractures.

Timing of Theatres

During the pre-COVID-19 cohort (2019/2020), a total of 397 surgeries were undertaken, with 61.7% of them adhering to the stipulated 36-hour punctuality threshold. In contrast, the COVID-19 cohort (2020/2021) witnessed 366 surgeries, of which 70.8% met the same punctuality criteria. This marked a notable 9.1% increase in punctuality during the COVID-19-affected year.

Remarkably, COVID-19 had a limited direct impact on surgery delays, as only 2.8% of cases were attributed to COVID-19-related delays. The primary cause of delay across both cohorts was identified as "administrative/logistic, cancelled due to list over-run," accounting for 46.7% of delays in 2019/2020 and increasing to 55.1% in 2020/2021. This heightened occurrence of delays due to list over-runs, despite a reduced surgical caseload, suggests that a decrease in the number of theatres did not affect improved punctuality. Instead, this phenomenon can be attributed to a broader reduction in other causes of delay, for example, "awaiting orthopedic diagnosis/investigation."

**Figure 2:**
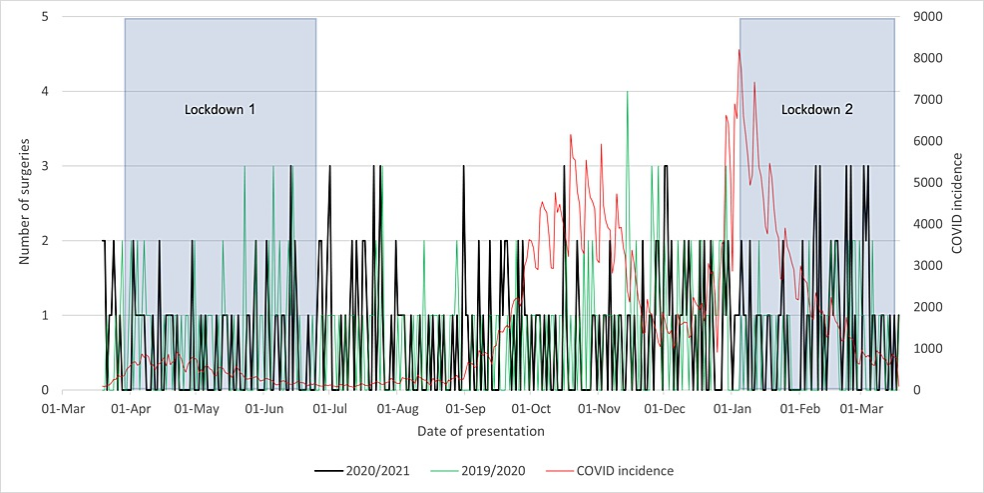
Cases of neck of femur fractures operated on within 36 hours in 2020/2021 compared to that in 2019/2020 and to the incidence of COVID-19 cases and lockdowns

**Figure 3:**
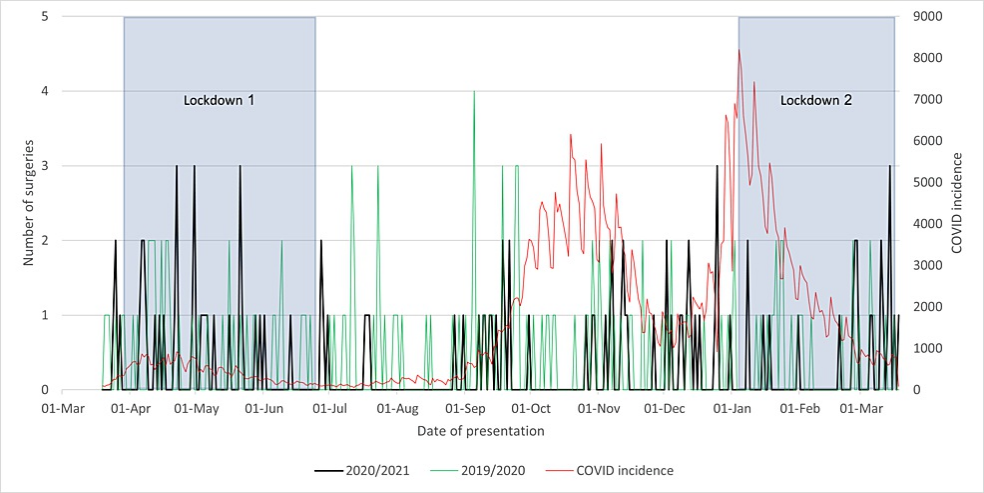
Cases of neck of femur fractures with delayed surgery (>36 hours) in 2020/2021 compared to that in 2019/2020 and to the incidence of COVID-19 cases and lockdowns

As seen in Figure 2, the improvement in punctuality following the initial lockdown, when COVID-19 incidence was low, may be attributed to regional public sentiment regarding the virus. Lower incidence rates resulted in reduced admissions of COVID-19-positive patients, but paradoxically, fewer individuals sought health services during the pandemic due to COVID-19 concerns. This created an environment in which health services across all specialties in the hospital experienced reduced strain, providing more time, resources, and attention to urgent procedures, such as surgeries for hip fractures (NoF). A further examination of Figure 3 reveals distinct spikes during the initial lockdown period, surpassing anticipated levels. These spikes, potentially linked to increased demands for hospital resources, shed light on the pandemic's influence on surgical punctuality. After the initial lockdown, a modest correlation emerged between delayed theatre frequency and COVID-19 incidence, particularly leading up to the peak in COVID-19 cases on January 4.

Patient Mobilization

**Figure 4:**
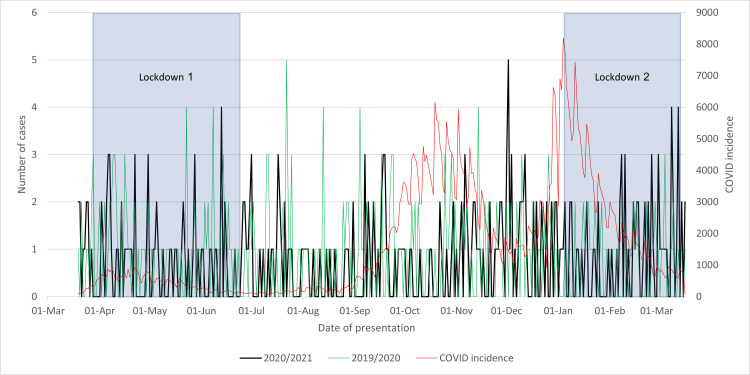
Cases of neck of femur fractures where postoperative mobilization was done within the same day in 2020/2021 compared to that in 2019/2020 and to the incidence of COVID-19 cases and lockdowns

**Figure 5:**
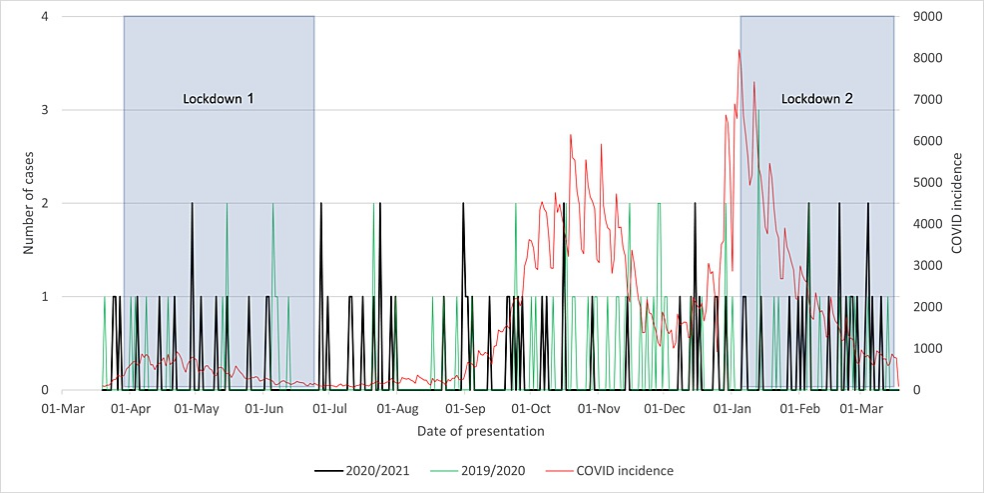
Cases of neck of femur fractures where postoperative mobilization was delayed in 2020/2021 compared to that in 2019/2020 and to the incidence of COVID-19 cases and lockdowns

As seen in both Figure 4 and Figure 5 about the mobilization of patients following their surgery, the 2020/2021 cohort followed the 2019/2020 trajectory far more closely than the COVID-19 incidence. Additionally, the lockdowns had no apparent effect. There was an overall decrease in delayed mobilization of 2% between the years, but such a small figure is unlikely to hold significance. These graphs exhibit no clear relation between COVID-19 and the mobilization of patients in the northwest center.

Patient Mortality

**Figure 6:**
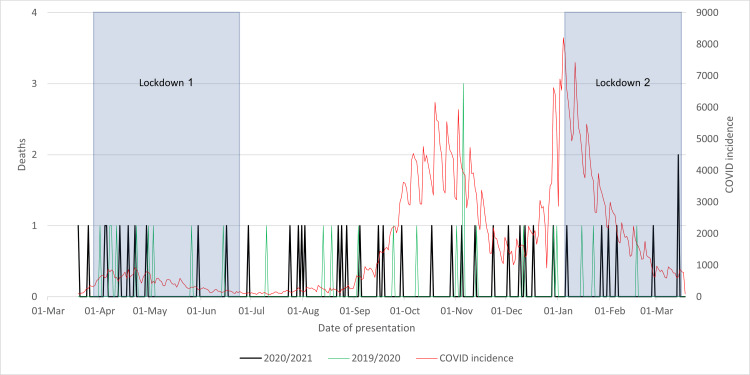
Mortality rate of neck of femur fractures while in-patient in 2020/2021 compared to that in 2019/2020 and to the incidence of COVID-19 cases and lockdowns

The 2020/2021 cohort exhibited a noteworthy 50% increase in hospital mortality when compared to the 2019/2020 cohort. As seen in Figure 6, these fatalities were uniformly distributed across the year and displayed no apparent correlation with lockdown measures or COVID-19 incidence rates. In contrast to expectations stemming from a reduced incidence of hip fractures (NoF fractures), a decline in mortality rates was not observed. This anomaly suggests that the cause of these deaths is more likely associated with deficiencies in healthcare staff rather than patient-related activities. The pandemic imposed considerable strain on healthcare workers due to increased workloads, evolving protocols, and constrained resources, potentially resulting in a compromised quality of care and subsequently elevating hospital mortality rates.

**Figure 7:**
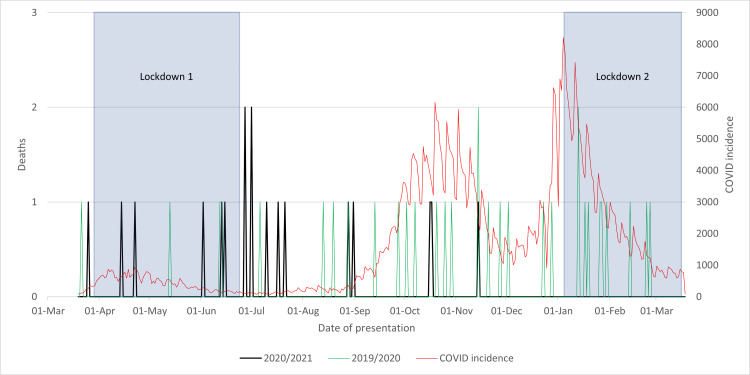
Mortality rate of neck of femur fractures within 120 days after discharge in 2020/2021 compared to that in 2019/2020 and to the incidence of COVID-19 cases and lockdowns

As seen in Figure 7, initially, the post-discharge mortality trend resembled that of hospital mortality, with the 2020/2021 cohort experiencing more deaths than their 2019/2020 counterparts. However, after the first lockdown was lifted, a marked spike occurred in the 2020/2021 cohort, with seven deaths recorded in the subsequent month, as opposed to a single death in the 2019/2020 cohort. Subsequently, the 2020/2021 cohort's mortality decreased, with only three deaths observed from August onward, a pattern not observed in the previous year. Overall, there was a 54.2% reduction in mortality throughout the year.

This shift in mortality rates may be attributed to the resumption of risk-taking behaviors and activities detrimental to health following the easing of lockdown restrictions. With the reinstatement of non-essential activities, it is conceivable that some patients engaged in behaviors such as excessive alcohol consumption or strenuous physical activity, potentially leading to further medical complications, especially in individuals with a history of joint fractures. Subsequent to this peak, few residential mortalities were noted, indicating that measures implemented by care homes, including the use of personal protective equipment, social distancing, symptom monitoring, and the early vaccination of residents, effectively mitigated mortality following discharge. This is corroborated by the decline in the mean age of mortality from 87 in 2019/2020 to 85 in 2020/2021, signifying improved care for the very elderly, a population predominantly residing in care homes.

Evaluating the effect of COVID-19 incidence and lockdowns on 2020/2021 NoF fracture demographics

Age

**Figure 8:**
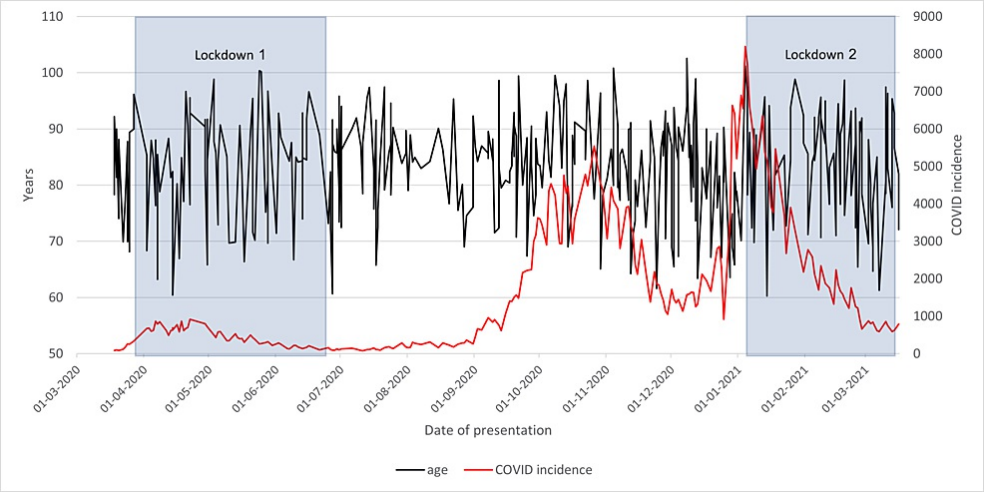
Age of the presentation of neck of femur fracture cases in 2020/2021 compared to the incidence of COVID-19 cases and lockdowns

Patient age varies from 61 years to 103 throughout the year. By plotting a trend line as in Figure 8, it is evident that the mean age of presentation did not vary with time; however, the age of presentation plot became more erratic following the increase in COVID-19 incidence during October.

This phenomenon could be due to COVID-19 contributing to frailty. NoF fractures are often preceded by falls, a significant risk factor for frailty. Frailty can be caused by existing comorbidities or illness. Therefore, with more of the population contracting COVID-19, more of the population is at risk of developing NoF fractures. This would impact both older and younger patients in the local region alike, causing no variation in the mean age of presentation.

Patient Sex

**Figure 9:**
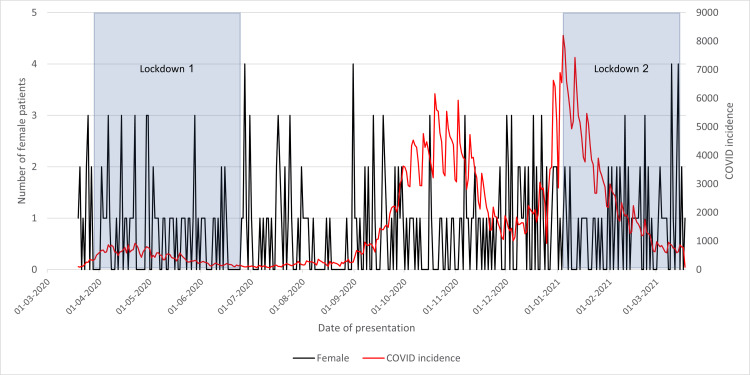
Female patients presenting with neck of femur fracture in 2020/2021 compared to the incidence of COVID-19 cases and lockdowns

**Figure 10:**
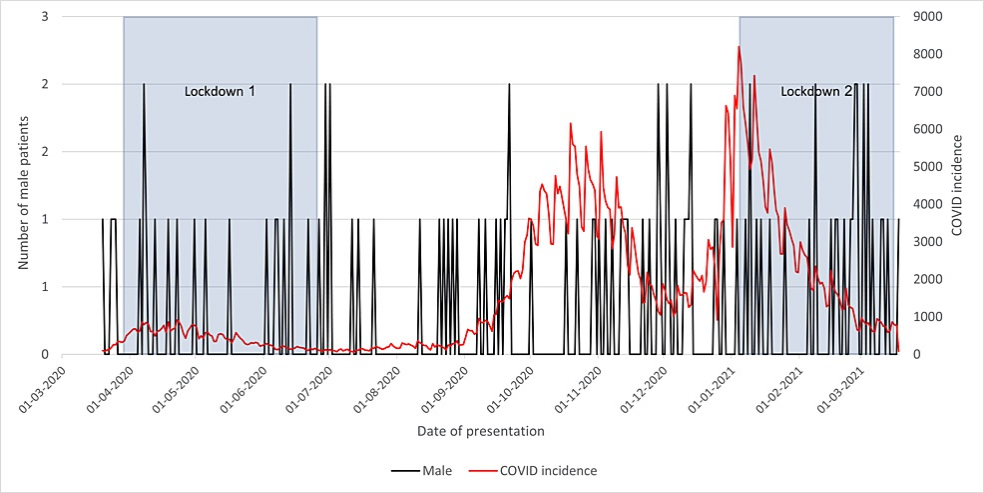
Male patients presenting with neck of femur fracture in 2020/2021 compared to the incidence of COVID-19 cases and lockdowns

As seen in Figure 9 and Figure 10, both female and male patients presented reasonably consistently throughout the year, with no noticeable temporal variations. Therefore, neither COVID-19 nor lockdowns had any impact on either gender differently.

Time From Presentation to Admission

**Figure 11:**
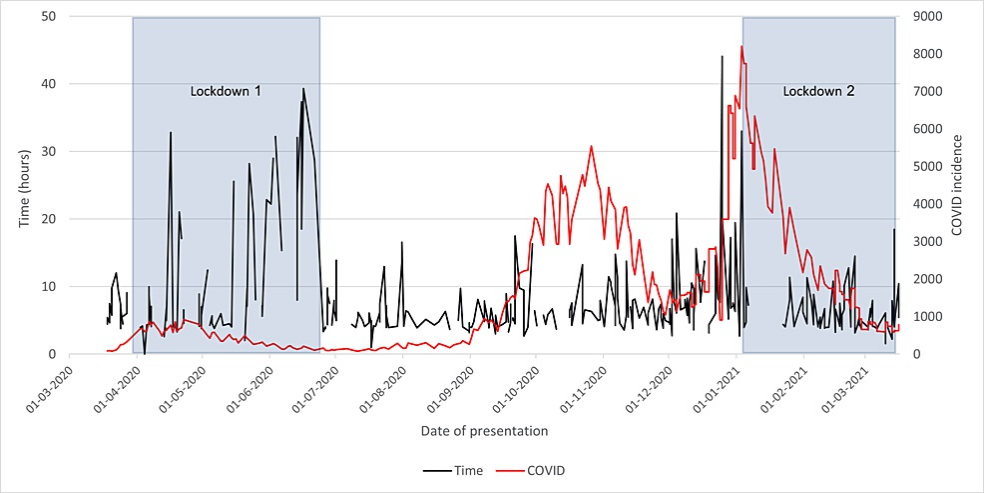
Time from the presentation of neck of femur fracture patients to admission in 2020/2021 compared to the incidence of COVID-19 cases and lockdowns

Initially, as seen in Figure 11, the plot fluctuates, peaking several times. However, after lifting lockdown restrictions, the time taken significantly reduces and stabilizes. The time taken remains fairly consistent until late December when it begins to peak again, coinciding with COVID-19 incidence. Vital data in early-mid January was not recorded; however, when the plot resumes, the time has once again reduced and stabilized.

This graph depicts that the first lockdown was ineffective at reducing the time taken in Wythenshawe Hospital from presentation to admission; however, the second lockdown may have improved this. This could be caused by physician unfamiliarity with the altered patient pathways introduced during lockdown. Many local guidelines were changed within the hospital regarding patient isolation, the use of aerosol-generating procedures, and other aspects; initially, healthcare workers would not be accustomed to these, resulting in a delay in admitting patients. However, by the time the second lockdown was introduced, as these measures had previously been introduced, the healthcare workers had a far better understanding of these changes, resulting in faster admission.

Time From Presentation to Surgery

**Figure 12:**
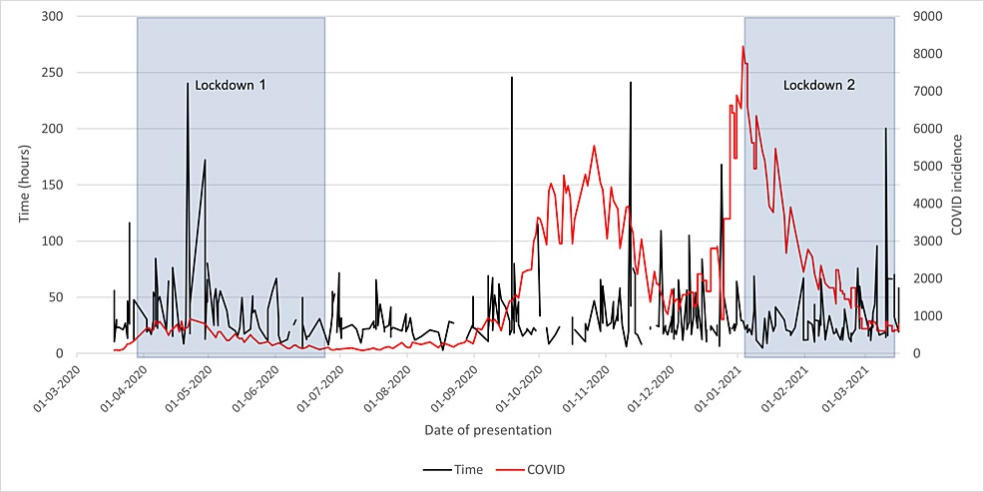
Time from the presentation of neck of femur fracture patients to surgery in 2020/2021 compared to the incidence of COVID-19 cases and lockdowns

Figure 12 shows that immediately prior to the introduction of the second lockdown, the COVID-19 incidence rate was at its highest; at the same point in time, the time taken from the presentation of a NoF fracture to theatre had been comparatively quick.

Overall, the graph shows no clear pattern between COVID-19 cases and the time until surgery. Several peaks are seen throughout the year; however, no association is seen with either lockdowns or COVID-19. It is fair to conclude that COVID-19 has no clear impact on the time taken from presentation to surgery in this center.

Time From Presentation to Discharge

**Figure 13:**
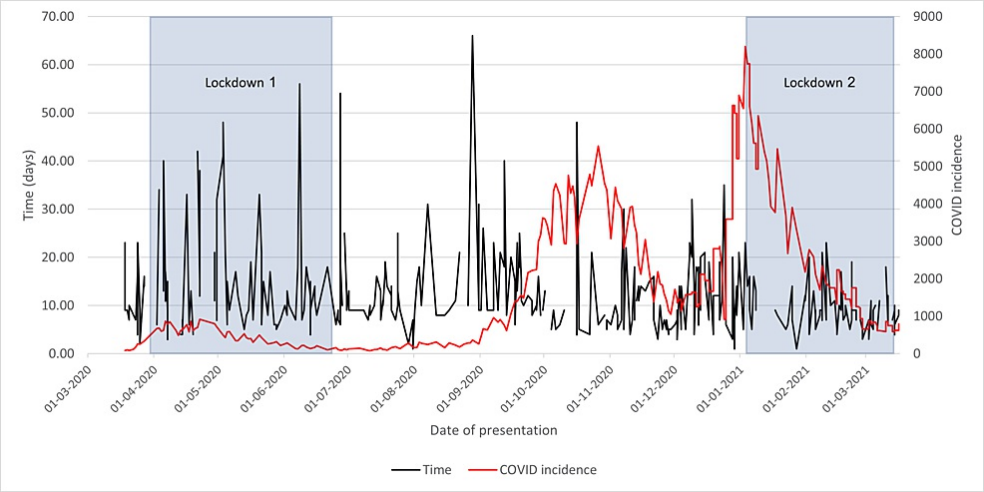
Time from the presentation of neck of femur fracture patients to discharge in 2020/2021 compared to the incidence of COVID-19 cases and lockdowns

Much like the time from presentation to admission graph (Figure 11), Figure 13 shows an initial fluctuating plot until the termination of the first lockdown. However, unlike the other graphs, there is no clear correlation with COVID-19 incidence as the time taken remains consistently lower. The second lockdown succeeded in reducing the time taken and preventing peaks from occurring. These findings are likely also to be due to healthcare worker familiarity with altered patient pathway during periods of lockdown.

COVID-19-Positive Results on Admission to Wythenshawe Hospital

**Figure 14:**
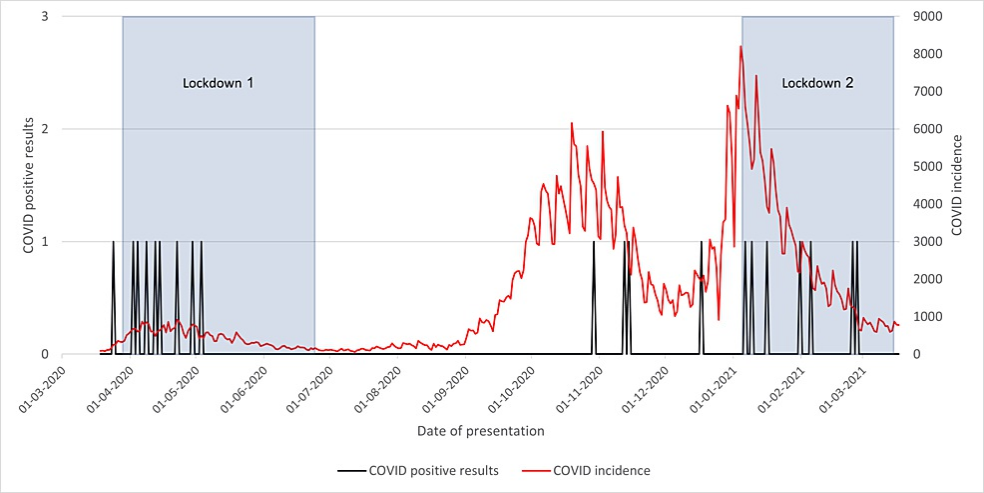
Cases with neck of femur fracture testing COVID-19-positive on admission in 2020/2021 compared to the incidence of COVID-19 cases and lockdowns

Initially, regular positive results were found despite the initiation of the first lockdown, until the last recorded case on 3 May 2020, after which no positive results were identified for over five months. As evidenced in Figure 14, as COVID-19 incidence increased, positive results on admission became more regular and persisted throughout the second lockdown.

These findings may be explained by public engagement with lockdowns. During the first lockdown, the public engaged with measures strictly. A slight latency could explain the initial cases, as COVID-19 can be contracted and remain asymptomatic for a period before presentation; halfway through this lockdown, the positive results stopped for a period. However, the same was not seen for the second lockdown. This could be due to public disillusion and the loss of enthusiasm; people followed the preventative measures far more loosely when compared to the first lockdown, causing increased contraction in patients who then went on to present.

COVID-19-Positive Results After Theatre

**Figure 15:**
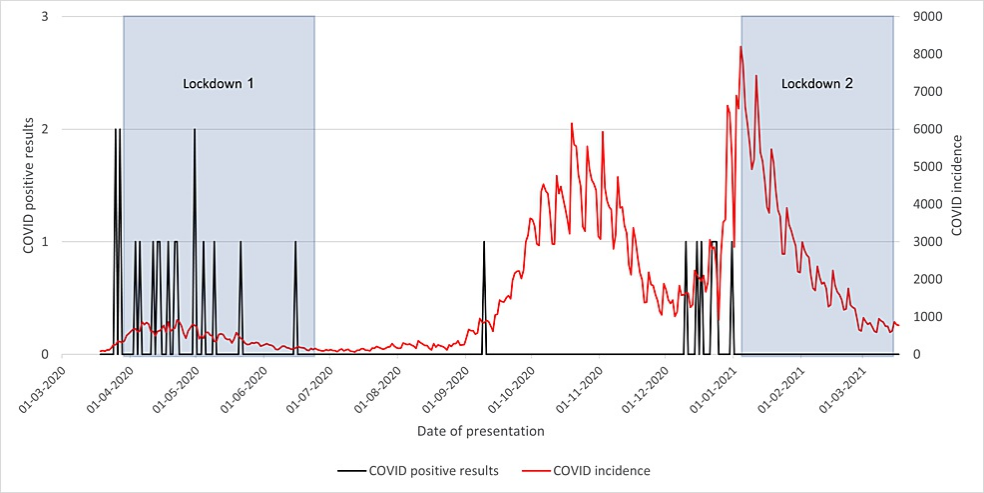
Cases with neck of femur fracture testing COVID-19-positive postoperatively in 2020/2021 compared to the incidence of COVID-19 cases and lockdowns

Much like the previous graph (Figure 14), in Figure 15, many cases were seen during the first lockdown. However, upon lifting these measures, positive results appeared to cease until 9 September 2020. No further cases were seen during the November spike, but more positive cases were seen in the lead-up to the January spike. Despite this, during the second lockdown, no positive cases after theatre were recorded.

This could be explained again by healthcare workers' familiarity with altered patient pathways, as seen in time from presentation to admission and discharge. During the first lockdown, healthcare workers were not accustomed to new changes that had been implemented for COVID-19-positive patients, allowing for transmission during admission to the hospital, resulting in nosocomial infection. However, by the second lockdown, they will be less likely to make the same mistakes, resulting in minimal transmission within the center.

Death of COVID-19-Positive Patients in Hospital

**Figure 16:**
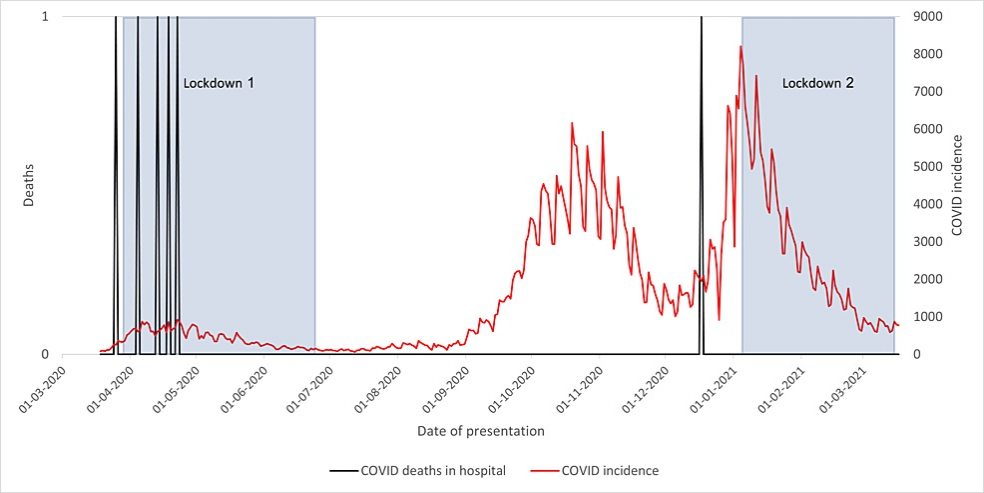
Deaths of neck of femur fracture patients due to COVID-19 in 2020/2021 compared to the incidence of COVID-19 cases and lockdowns

In Figure 16, cases are seen in the early months, with one further case seen in December. While not obviously coherent with the COVID-19 incidence plot, it follows the COVID-19-positive result graphs.

This indicates that community COVID-19 has little impact on COVID-19-positive NoF fracture patient mortality in Wythenshawe Hospital; cases are seen at times when more hospital patients are found positive, rather than when the population incidence is high.

## Discussion

On exploring the intricate relationship between COVID-19 and the care of patients with NoF fractures within the Wythenshawe Hospital orthopedic department, most parameters remained largely unaffected by the pandemic itself. The data generated from our study suggests that COVID-19 incidence has only a modest association with NoF fracture patients, with correlations primarily observed in patient age and the time elapsed from presentation to admission.

The graphical representations in our study underscore the substantial impact of regional lockdown measures on patient care. The initial lockdown appeared effective in reducing the number of COVID-19-positive cases upon admission, possibly due to the rigorous adherence of the public to restrictions in the northwest region. However, adverse consequences became evident during these lockdown periods, including prolonged intervals from presentation to admission and discharge, along with surgical delays. These challenges could be attributed to heightened demands on healthcare resources, as well as the adjustment period required for hospital staff to become familiar with new COVID-19-related protocols.

Conversely, the second lockdown witnessed a reversal of these trends, as the time from presentation to admission and discharge improved, and no COVID-19-positive cases were recorded post-surgery, with no fatalities following discharge. This improvement may be attributed to lessons learned from the initial lockdown, resulting in increased local resource allocation and greater familiarity with hospital-specific lockdown measures.

While our study is limited to patients at Wythenshawe Hospital, its implications extend beyond this single institution. The insights gleaned from our data could be extrapolated to inform healthcare practices across the entire United Kingdom, especially in anticipation of future waves of the pandemic [16]. Our findings suggest that most parameters are likely to remain stable, but a potential decrease in NoF fracture incidence, greater variability in patient age, and extended timeframes from presentation to surgery may be expected. Furthermore, we anticipate that additional lockdowns may yield diminishing returns as public compliance wanes, potentially exacerbating the negative consequences observed, such as increased COVID-19-positive admissions, greater age variation, and elevated mortality rates post-discharge. By anticipating these trends, preemptive measures can be implemented to mitigate their impact.

While the United Kingdom is progressing toward the end of the pandemic with widespread vaccine distribution, it is imperative to recognize that many other countries may not have the same resources to provide vaccination to their populations promptly. Consequently, they may grapple with the virus for an extended duration. The findings from this study, conducted in a single center, can be extrapolated and applied in these nations to anticipate how NoF fracture patient care may be affected by the pandemic and associated preventive measures.

Limitations

This study has several limitations that need to be addressed. Primarily, we only used data from one specific medical center, so the trends we found may not apply to other regions in the United Kingdom or other countries. It would be valuable to expand the study to include data from different places to make our findings more widely applicable.

Certain gaps in data remained since data was collected retrospectively from an audit database. Having more complete data would have made more continuous graphs pointing toward any missed trends.

Moreover, we could have gained a better understanding of how COVID-19 affected these patients by considering additional factors. For example, we could have included a measure of their quality of life, which would have given us a more complete picture of their well-being during and after their experience with the virus.

Lastly, to make our results more reliable, we could have used more rigorous statistical tests. These tests would have helped us determine whether the patterns we observed were statistically significant. However, we have used graphical patterns to determine trends and are not drawing causality in this study. It would be something for future studies with larger datasets to explore and draw stronger correlations from.

## Conclusions

In conclusion, the COVID-19 pandemic has exerted a profound and multifaceted influence on various facets of our lives. This study has explored its potential ramifications on neck of femur (NoF) fracture patients, acknowledging the scarcity of comprehensive research on this subject and the incongruities present in the existing literature. Leveraging data from the National Hip Fracture Audit and focusing on Wythenshawe Hospital as a microcosm, this investigation sheds light on the intricate relationship between the pandemic and the care of NoF fracture patients. The findings reveal a dynamic interplay between COVID-19 incidence and NoF fracture rates, with increasing national COVID-19 cases coinciding with decreased NoF fracture incidence. Notably, this period witnessed enhancements in theatre punctuality and reduced hospital mortality, greater age diversity among patients, prolonged intervals between presentation and admission and discharge, and an elevated prevalence of COVID-19-positive test results at admission and post-surgery within the hospital.

Additionally, the study underscores the significant influence of lockdown measures on patient care. The initial lockdown correlated with a rise in COVID-19-positive test results at admission but posed challenges in terms of delayed surgeries and extended timeframes for admission and discharge, leading to a spike in post-discharge mortality. Conversely, the second lockdown demonstrated improved efficiency in terms of timing and safety, as evidenced by expedited processes, the absence of COVID-19-positive results following surgery, and zero post-discharge fatalities. These findings collectively highlight the intricate relationship between the COVID-19 pandemic and the care of NoF fracture patients, emphasizing the need for further research and tailored strategies to optimize patient outcomes during pandemics and associated lockdowns.
